# Full-length transcriptome reveals the pivotal role of ABA and ethylene in the cold stress response of *Tetrastigma hemsleyanum*

**DOI:** 10.3389/fpls.2024.1285879

**Published:** 2024-01-31

**Authors:** Lihua Qian, Shuya Yin, Na Lu, Erkui Yue, Jianli Yan

**Affiliations:** ^1^ Institute of Biotechnology, Hangzhou Academy of Agricultural Sciences, Hangzhou, China; ^2^ Institute of Vegetable, Hangzhou Academy of Agricultural Sciences, Hangzhou, China; ^3^ Institute of Crop Science and Ecology, Hangzhou Academy of Agricultural Sciences, Hangzhou, China

**Keywords:** *Tetrastigma hemsleyanum*, full-length transcriptome, cold stress, ABA, ethylene

## Abstract

*Tetrastigma hemsleyanum* is a valuable herb widely used in Chinese traditional and modern medicine. Winter cold severely limits the artificial cultivation of this plant, but the physiological and molecular mechanisms upon exposure to cold stress in *T. hemsleyanum* are unclear. *T. hemsleyanum* plants with different geographical origins exhibit large differences in response to cold stress. In this research study, using *T. hemsleyanum* ecotypes that exhibit frost tolerance (FR) and frost sensitivity (FS), we analyzed the response of cottage seedlings to a simulated frost treatment; plant hormones were induced with both short (2 h) and long (9 h) frost treatments, which were used to construct the full-length transcriptome and obtained 76,750 transcripts with all transcripts mapped to 28,805 genes, and 27,215 genes, respectively, annotated to databases. Kyoto Encyclopedia of Genes and Genomes (KEGG) pathway analysis showed enrichment in plant hormone signaling pathways. Further analysis shows that differently expressed genes (DEGs) concentrated on calcium signaling, ABA biosynthesis and signal transduction, and ethylene in response to cold stress. We also found that endogenous ABA and ethylene content were increased after cold treatment, and exogenous ABA and ethylene significantly improved cold tolerance in both ecotypes. Our results elucidated the pivotal role of ABA and ethylene in response to cold stress in *T. hemsleyanum* and identified key genes.

## 1 Introduction

*Tetrastigma hemsleyanum* Diels et Gilg is an herbaceous climber that is widely distributed in tropical and subtropical regions, mainly in provinces of south and southwest China (Wang et al., 2015). In China, *T. hemsleyanum* is used as traditional medicine “San ye qing,” a broad-spectrum antibiotic material against fever and inflammation (Li, 1998). It has also exhibited antioxidant, antivirus, antitumor, immunomodulatory, and hypoglycemic effects in modern pharmacological research studies (Ji et al., 2021). Because of its slow growth, it usually takes 3–5 years to meet the requirements of commercial medicinal materials, so it is a precious perennial medicinal resource. Due to overexploitation, wild resources have been on the verge of extinction in recent years (Peng et al., 2019) and the supply of medicinal herbs is largely dependent on cultivation. For synthesized utilization of land and imitating wild conditions to improve the quality, artificial *T. hemsleyanum* is usually planted in the mountains, causing the herb to suffer from winter cold frequently.

Cold stress is a major environmental factor that limits agricultural production (Einset et al., 2007). Low temperatures limit CO_2_ fixation coupled with an overreduction in the electron transport chain, leading to photosynthesis suppression and dramatic increases in reactive oxygen species (ROS) (Maleki and Ghorbanpour, 2018). In response to abiotic stresses, plants have evolved highly complex adaptive mechanisms. Once the receptor is triggered by cold stimulation, a signal is spread by transduction pathways, and induced gene-expression changes subsequently cause physiological changes, including an increase in antioxidant levels, induction of influx of cellular calcium ions, alteration in membrane lipid composition, adjustment of hormone levels, and changes in electrolyte leakage and soluble proteins.

Low temperatures, especially winter minimum temperatures, are one of the primary forces determining the geographic boundaries of many plant species (Pither, 2003), but physiological tolerance to cold also varies among populations within a species (Armstrong et al., 2020). *T. hemsleyanum* plants with different geographical origins contained enormous genetic variability (Peng et al., 2015), which caused different morphological characteristics and environmental adaptability. It provides convenience for ascertaining the cold response and regulatory networks of cold tolerance in *T. hemsleyanum*.

Short-read RNA-Seq (mainly using Illumina technology) has been used for over a decade; however, Illumina sequencers are appropriate only for short-read-length sequencing because of information loss caused by fragments during sample preparation and spliced after sequencing (Cui et al., 2020). Nanopore sequencing technology is a new approach that can sequence single long DNA and RNA molecules (Deamer et al., 2016; Wang Y. H. et al., 2021). Since the first nanopore sequencer, MinION, was provided by Oxford Nanopore Technologies (ONT), it has been used extensively to assemble the initial reference genomes of many non-model organisms (Scheunert et al., 2020), but only several researchers in transcriptomics have explored the molecular mechanisms in plants to date, such as in *Arabidopsis* (Cui et al., 2020; Parker et al., 2020; Qin et al., 2022), rose (Li et al., 2021), peach (Liu et al., 2021), quinoa (Zheng et al., 2022), and potatoes (Xiong et al., 2022).

In this research study, we monitored the phenotypes of *T. hemsleyanum* seedlings at their preferred temperature, 2 h and 9 h frost stress, and sequenced their full-length transcripts using the ONT method. As a result, we identified numerous genes that may take part in the cold stress response. These genes are involved in hormone and signal transduction pathways, especially the biosynthesis and signal transduction of ABA and ethylene. Endogenous ABA and ethylene contents were increased after cold treatment, and exogenous ABA and ethylene significantly improved cold tolerance. These results indicated the pivotal role of ABA and ethylene in the cold stress response of *T. hemsleyanum.* This study provides insight into the cold stress response pathway in *T. hemsleyanum* and thus provides a basis for improving the resistance of this cash crop to cope with increasingly frequent cold waves in the major grain-producing area south of the Yangtze River in China.

## 2 Results

### 2.1 Variations in cold tolerance of different *T. hemsleyanum* ecotypes

**Figure 1:**
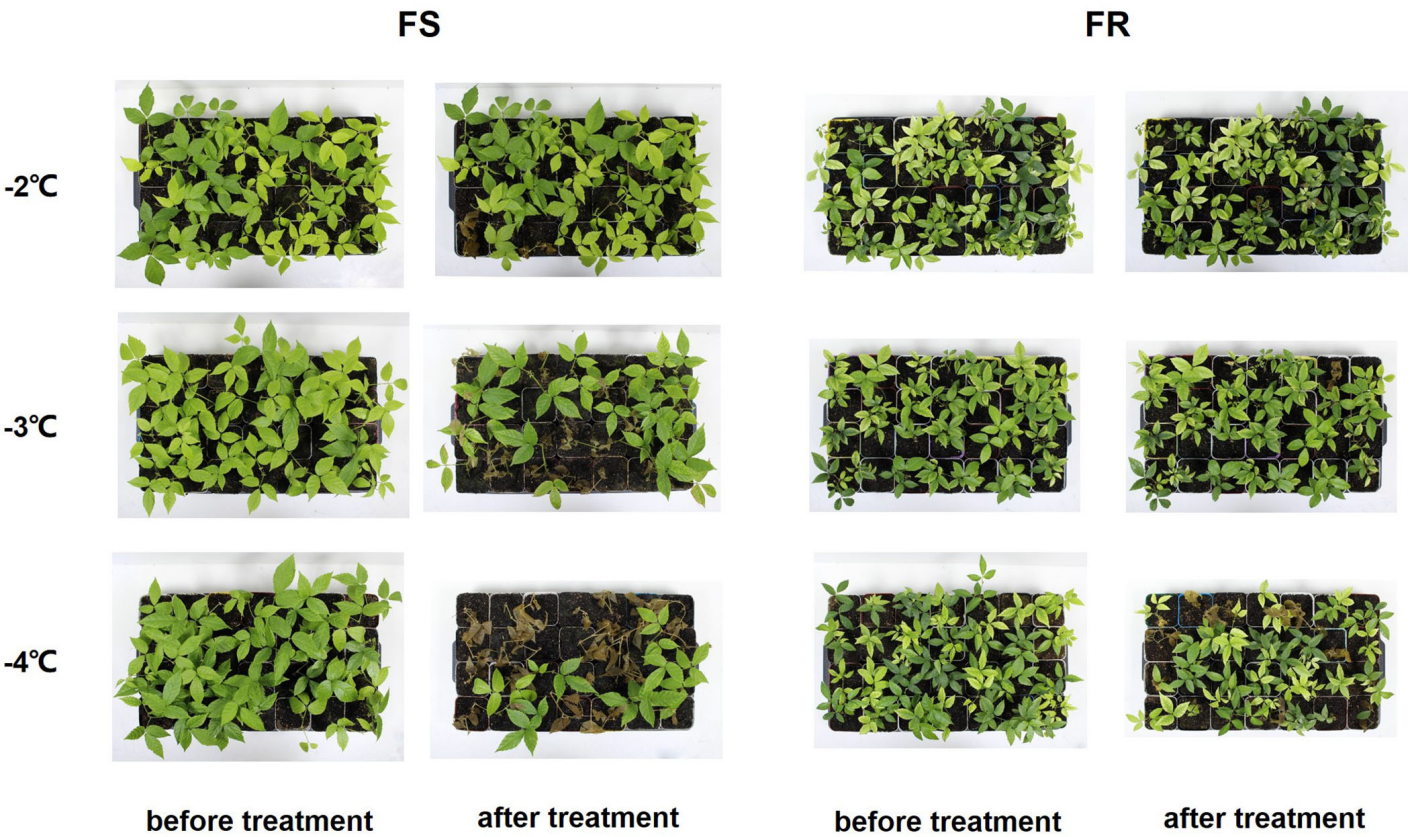
Cold injury in different *T. hemsleyanum* cottage ecotypes. Representative images of 2-month-old cottage seedlings of FR and FS grown under control (CK) and exposed to a simulated frost treatment for 12 h (T) were exhibited to demonstrate the difference in frost tolerance between the two ecotypes.

*T. hemsleyanum* collected from different geographical areas exhibits various cold tolerances. When exposed to −3°C, the leaves of sensitive *T. hemsleyanum* ecotype seedlings exhibited severe damage; most sensitive ecotype seedlings died after exposure to −4°C for 12 h (**Figure 1**). It is difficult to take typical tissue for further analysis. Therefore, in this study, cottage seedlings of *T. hemsleyanum* ecotypes that exhibited frost tolerance (FR) and frost sensitivity (FS) were selected and exposed to a simulated frost treatment (−2°C) for analysis of physiological indicators and gene expression. In contrast, a −4°C simulated frost treatment was performed to evaluate the cold tolerance survival rate.

### 2.2 ONT RNA-seq and functional annotation

To investigate the molecular mechanisms underlying the frost response, transcriptome sequencing was performed on the leaves of *T. hemsleyanum* ecotype seedling growth at normal temperature (25°C, CK), exposed to frost conditions (−2°C) for a short time (2 h) and a long time (9 h).

A total of 18 libraries from six samples (three biological replicates per sample) were obtained. The clean data output from each sample was at least 6 GB, and 89%~92% of clean reads, namely, 5.40–8.95 million, were full-length reads. After mapping to the *T. hemsleyanum* genome, 4.83~7.94 million reads were mapped, and mapped rates in all libraries exceeded 97% (**Supplementary Table 1**).

**Figure 2:**
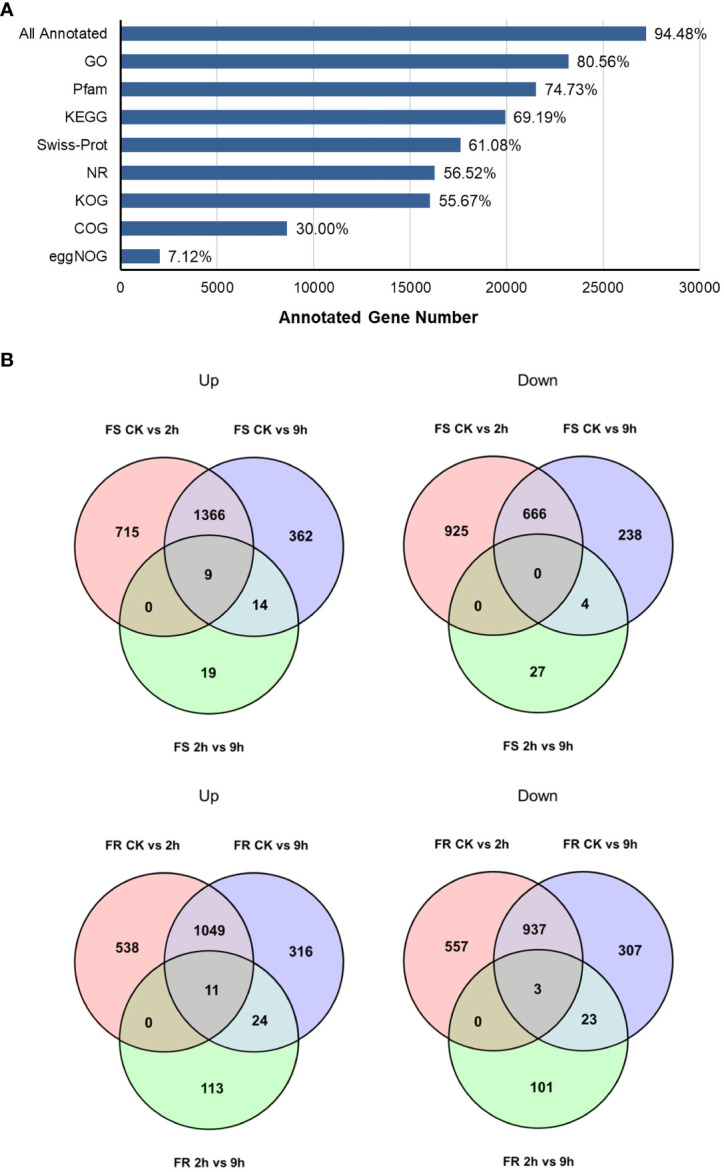
DEGs in *T. hemsleyanum* in response to cold stress. **(A)** Functional annotations of all genes in the indicated databases. **(B)** The number of DEGs induced and reduced by cold.

A total of 76,750 transcripts were obtained, and 53,190 transcripts were newly found. A total of 28,805 transcripts were mapped to existing genes, and 4,121 were new genes (**Figure 2A**). A total of 27,215 genes were annotated in databases, and the percentage that was annotated was 94.5% (**Figure 2A**).

### 2.3 Kyoto Encyclopedia of Genes and Genomes clustering analysis of differently expressed genes

To further elucidate the changes regulated by cold stress in two *T. hemsleyanum* ecotypes with different cold tolerances, we analyzed the differently expressed genes (DEGs) in FS and FR after cold treatment for 2 h and 9 h relative to CK, respectively, and DEGs in cold treatment for 9 h relative to 2 h. DEGs were identified based on a false discovery rate (FDR) ≤0.05 and fold change ≥2. A total of 6,086 DEGs were detected in all the 18 cDNA libraries: 3,683 genes were differentially expressed after 2 h and 3,095 after 9 h of cold stress exposure in FS, and 2,659 genes were differentially expressed after 2 h and 2,670 after 9 h of cold stress exposure in FR (**Figure 2B**). Generally speaking, the 9-h-simulated frost treatment resulted in more DEGs, but most of these were already present after 2 h, with only 73 DEGs in FS and 275 DEGs in FR differentially expressed after 9 h of cold treatment relative to 2 h, which may suggest a rapid response of transcriptomic changes, which already happened a short time after applying cold stress. There were more DEGs in FS than in FR compared with their respective controls, indicating that some adaptive traits already exist in the more cold-tolerant FR at moderate temperature conditions, resulting in FR being less affected and the sensitive variety FS being more affected by cold stress.

**Figure 3:**
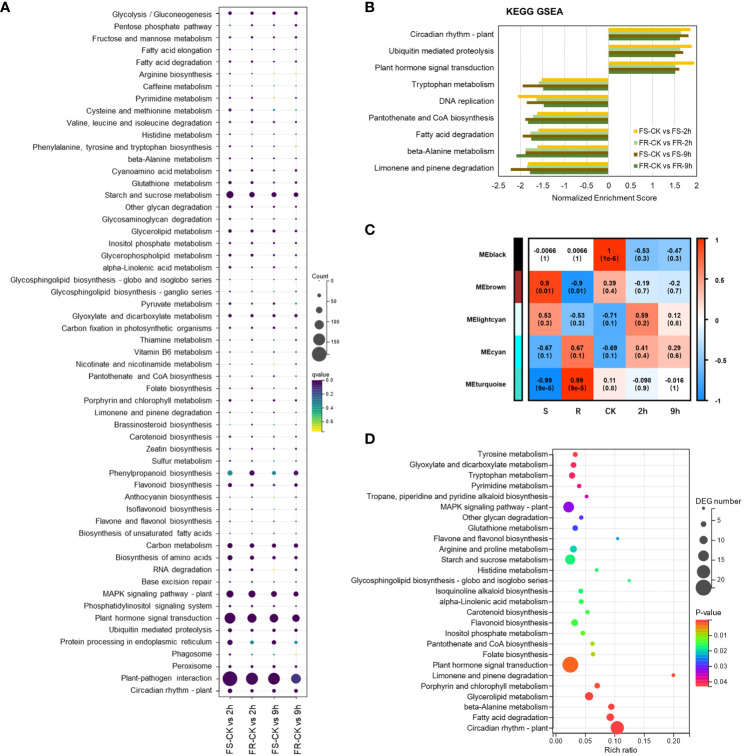
KEGG clustering analysis of DEGs. **(A)** KEGG pathway enrichment analysis based on DEGs. **(B)** KEGG pathway enrichment analysis by GSEA. **(C)** Relationships between module eigengenes and external traits in WGCNA. Each row represents a consensus module, and each column represents a specific trait. The numbers in each grid represent the correlation coefficients between module eigengenes and corresponding traits, with P values indicated in parentheses below. **(D)** KEGG pathway enrichment analysis based on DEGs in the black module (P < 0.05).

KOBAS 2.0 software was used for the Kyoto Encyclopedia of Genes and Genomes (KEGG) enrichment analysis of DEGs in 2-h and 9-h treatment samples relative to controls in both ecotypes. DEGs were annotated to 134 KEGG reference pathways (**Figure 3A**), 26 KEGG pathways induced by cold were predominantly significantly enriched in at least one dataset, and 58 KEGG pathways suppressed by cold were predominantly significantly enriched in at least one dataset (**Supplementary Figure 1**).

DEGs participating in inositol phosphate metabolism (ko00562), glyoxylate and dicarboxylate metabolism (ko00630), MAPK signaling pathway (ko04016), plant hormone signal transduction (ko04075), circadian rhythm (ko04712), and ubiquitin-mediated proteolysis (ko04120) were predominantly upregulated, and fatty acid degradation (ko00071), starch and sucrose metabolism (ko00500), other glycan degradation (ko00511), limonene and pinene degradation (ko00903), base excision repair (ko03410), beta-alanine metabolism (ko00410), pantothenate and CoA biosynthesis (ko00770), and peroxisome biosynthesis (ko04146) were predominantly downregulated after 2 h and 9 h of cold stress in both ecotypes.

To further elucidate the cold-induced transcriptomic variations, gene set enrichment analyses (GSEAs) were performed (**Figure 3B**). Plant hormone signal transduction (ko04075), circadian rhythm (ko04712), and ubiquitin-mediated proteolysis (ko04120) pathways were also induced. At the same time, fatty acid degradation (ko00071), limonene and pinene degradation (ko00903), beta-alanine metabolism (ko00410), pantothenate and CoA biosynthesis (ko00770), DNA replication (ko03030), and tryptophan metabolism (ko00380) were suppressed significantly in both ecotypes after the 2-h and 9-h treatments. It indicated that these pathways might be central mechanisms in response to low-temperature stress.

To better understand the correlation between these DEGs and the variation in the cold stress response, we performed a weighted correlation network analysis (WGCNA). Co-expression modules were generated by linking to the traits, including ecotypes and cold treatments. A total of five modules were generated (**Figure 3C**), and consistent with the pairwise comparison, modules colored black showed a significant correlation with normal atmospheric temperature, DEGs in black module obviously enrichment in plant hormone signal transduction (ko04075) (**Figure 3D**), but no modules correlating with the 2-h or 9-h cold treatments were found.

### 2.4 Temperature sensing in the cold response

Changes in membrane fluidity are widely considered to be involved in temperature sensing. Both cold and heat alter cellular membrane fluidity, which can affect the structure and/or activity of membrane-localized proteins such as Ca^2+^ channels, thereby triggering Ca^2+^ influx, a crucial process for inducing temperature-responsive gene expression.

**Figure 4:**
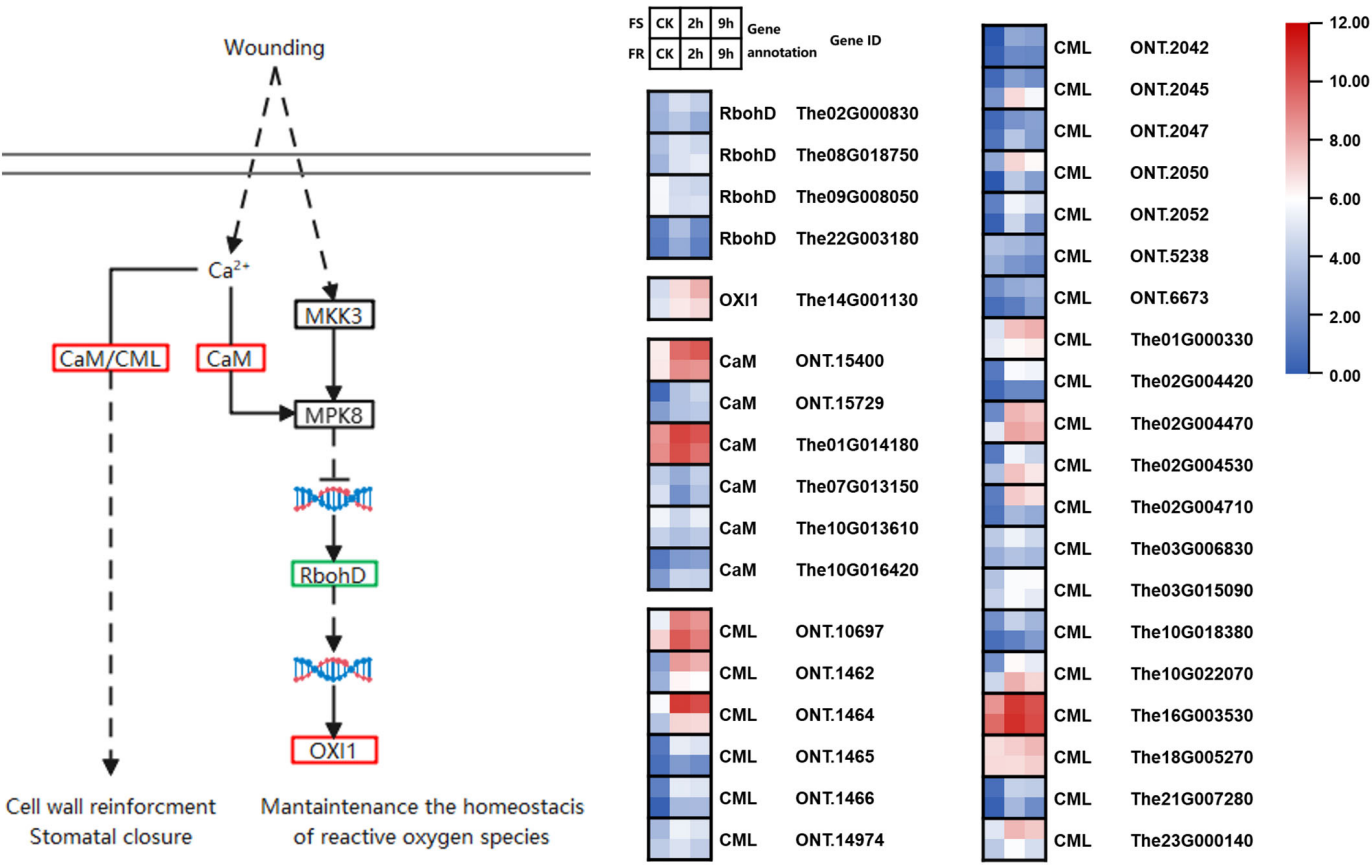
Cold-responsive DEGs involved in temperature sensing and calcium signal pathways. Each colored cell in the heatmap represents log2 of the DEG CPM (mean of three biological replicates); red represents induced genes, and blue represents repressed genes. A schematic diagram of signaling pathway genes; red boxes represent all annotated genes that were upregulated, blue boxes represent all annotated genes that were downregulated, green boxes represent both up and downregulated genes found in this location, and black boxes represent no DEGs found in this location.

In this study, we found six genes encoding calmodulin proteins, and four were induced by cold (**Figure 4**). There were two upregulated calmodulin proteins highly expressed; 1,621 counts of *The01G014180* were detected in samples at normal temperature in FS, significantly upregulated by 6.9-fold (11,123 counts) in the 2-h cold treatment and by 4.5-fold (7,294 counts) in the 9-h cold treatment. A total of 209 counts of *ONT.15400* were detected in CK, upregulated by 20.7-fold (43,293 counts) in the 2-h cold treatment and 31.2-fold (6,542 counts) in the 9-h cold treatment in FS. This may cause *OXI1* and three out of four *RbohD* genes to become upregulated in response to cold stress, especially *OXI1* (*The14G001130*) upregulated 6.5-fold after 2 h and 17.8-fold after 9 h of cold treatments in FS. These may be key genes in the response to cold stress.

These key genes were also induced in FR but slightly less than that in FS. A total of 2,034 counts of *The01G014180* were detected in CK samples, but it was upregulated by 4.3-fold (8,741 counts) in the 2-h cold treatment and by 1.86-fold (3,780 counts) in the 9-h cold treatment in FR. A total of 227 counts of *The01G014180* were detected in CK samples, upregulated by 8.73-fold (1,985 counts) in the 2-h cold treatment and by 7.36-fold (1,673 counts) in the 9-h cold treatment in FR. The expression of *ONT.15400* was 2.18- and 3.91-fold in FS compared with FR after 2-h and 9-h cold treatments. The same expression pattern also happened in *OXI1* (*The14G001130*).

In addition, 26 genes encoding calcium-binding protein (CML) were found, and 25 were induced by cold exposure. Most expressed lower, but three highly upregulated CML expression. Among them, *ONT.10697* and *The16G003530* were expressed more in FR than in FS at normal temperature and after cold stress, whereas *ONT.1464* was expressed more in FS. CML was found to regulate stress response through the nitrogen monoxide (NO) signaling pathway; these three CMLs may be key genes as well.

### 2.5 ABA biosynthesis and signaling pathways in cold response

Abscisic acid (ABA) is a vital phytohormone that plays a key role in plant stress responses. In this research study, DEGs were significantly clustered in plant hormone signal transduction, whereas many DEGs in ABA biosynthesis were upregulated; we speculated that ABA might be related to the *T. hemsleyanum* cold response.

**Figure 5:**
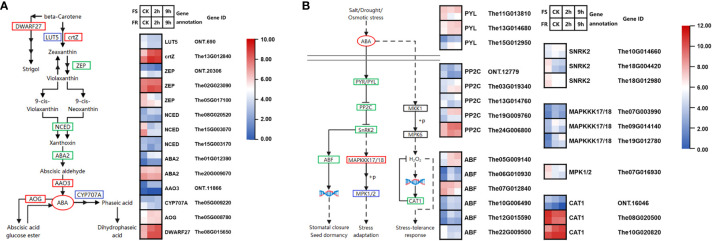
Cold-responsive DEGs regulate ABA biosynthesis and signaling pathways. **(A)** Cold-responsive DEGs in ABA biosynthesis. **(B)** Cold-responsive DEGs in the ABA signaling pathway. Each colored cell in the heatmap represents log2 of the DEG CPM (mean of three biological replicates); red represents induced genes, and blue represents repressed genes. A schematic diagram of signaling pathway genes; red boxes represent all annotated genes that were upregulated, blue boxes represent all annotated genes that were downregulated, green boxes represent both up and downregulated genes found in this location, and black boxes represent no DEGs found in this location.

There were 11 DEGs annotated to six KEGG catalogs in the ABA synthesis pathway, including *protein lutein deficient 5* (*LUT5, ONT.690*), *β-carotene 3-hydroxylase* (*crtZ, The13G012840*), *zeaxanthin epoxidase* (*ZEP, ONT.20306, The02G023090, and The05G017100*), *9-cis-epoxycarotenoid dioxygenase (NCED, The08G020520, The15G003070, and The15G003170*), *ABA DEFICIENT 2* (*ABA2*, *The01G012390*, and *The20G009070*), and *abscisic-aldehyde oxidase* (*AAO3*, *ONT.11866*). Most DEG levels were low or downregulated by cold, including three *NCED*s, which is the committing step in ABA synthesis; for instance, around 179 counts of *The15G003070 (NCED)* were detected in FS and 668 in FR at normal temperature, which was 3.7-fold that in FS, it was downregulated by 13.8-fold after 2 h and 7.2-fold after 9 h of cold stress in FS, and it was downregulated by 21.7-fold after 2 h and 20.2-fold after 9 h of cold stress in FR. However, the *crtZ* gene (*The13G012840*) and one *ZEP* gene (*The02G023090*) were highly expressed and upregulated by cold (**Figure 5A**).

Around 1,000 counts of *The02G023090* (*ZEP*) were detected in both ecotypes at normal temperatures. It was upregulated by 2.5-fold after 2 h and 2.0-fold after 9 h of cold stress in FR but not significantly upregulated in FS. As for *The13G012840* (*crtZ*), 1,130 counts were detected in FR at normal temperature, which was 4.7-fold that in FS. The expression of *The13G012840* was upregulated to 5,212 at 2 h and 3,977 at 9 h after cold treatments in FR, whereas it was also upregulated to 2,011 at 2 h and 2,752 at 9 h after cold treatments in FS. The13G012840 and The02G023090 may be key genes leading to the difference in cold tolerance between FR and FS.

*CYP707A* gene in the downstream ABA synthesis pathway transforms ABA to inactive metabolites such as phasic acid and dihydrophaseic acid; the CYP707A gene (*The05G009220*) was suppressed by cold in FR, whereas *abscisate beta-glucosyltransferase* (*AOG*), which controls ABA glycosylation for storage, was upregulated in both FS and FR, so that available ABA in plants increased.

In addition, the expression of *DWARF27* (*The08G01565*0), which controls the biosynthesis of strigol, was significantly upregulated and highly expressed. Strigol promotes the growth and nutrient uptake efficiency of inter- and endosymbiotic microorganisms, which may enhance the cold tolerance of plants in another way.

We found 17 DEGs annotated to four KEGG catalogs in the ABA signaling pathways (**Figure 5B**). *Protein phosphatase 2C* (*PP2Cs*) negatively regulated downstream genes, and ABA binds to *PYRABACTIN RESISTANCE 1 (PYR1*), which in turn binds to and inhibits PP2Cs. Two DEGs encoding *PYR/PYL* (*The13G014680* and *The15G012950*) and three DEGs encoding *PP2C* (*The03G019340*, *The19G009760*, and *The24G006800*) were upregulated by cold, especially *The24G006800* which was highly expressed and expressed more in FS, which may be the most critical DEG in the ABA signaling pathway.

There were two *SUCROSE NONFERMENTING 1-RELATED PROTEIN KINASE 2* genes (*SnRK2*, *The18G00442*0, and *The18G012980*) downregulated by cold, but finally, four *ABA-RESPONSIVE ELEMENT BINDING PROTEIN* genes (*ABF*, *The05G009140*, *The06G010930*, *The10G006490*, and *The12G015590*) were upregulated. In addition, *mitogen-activated protein kinase kinase kinase 17* (*MAPKKK17*) was induced by SnRK2 and positively regulated stress adaption; in this research study, three *MAPKKK17* (*The07G003990*, *The09G014140*, and *The19G012780*) were also upregulated by cold.

ABA can also regulate stress tolerance response through the MAPK pathway. There were two DEGs encoding *CAT* that were highly expressed and regulated by cold; the expression of *The08G020500* was suppressed, whereas *The10G020820* was induced by 4.4-fold at 2 h and 6.87 at 9 h in FS, whereas its expression was 5.7-fold in FR compared with FS; after cold stress, it reached more than 10,000 counts.

### 2.6 Ethylene biosynthesis and signaling pathways in cold response

The precursor of ethylene is produced from the methionine salvage pathway. Peroxides produced by cellular damage during stress were sensed. The signal was transduced to upregulate ethylene biosynthesis through the MAPK pathway. Two DEGs encoding mitogen-activated protein kinase (MPK3, *ONT.16948*, and *The19G006960*) were upregulated by cold; the expression of *The19G006960* was 3.7-fold higher in FR than in FS, and it was upregulated by 9.5-fold at 2 h and 6.4-fold at 9 h after cold treatment in FS. In contrast, it was upregulated by 2.8-fold at 2 h and 1.5-fold at 9 h after cold treatment in FR.

**Figure 6:**
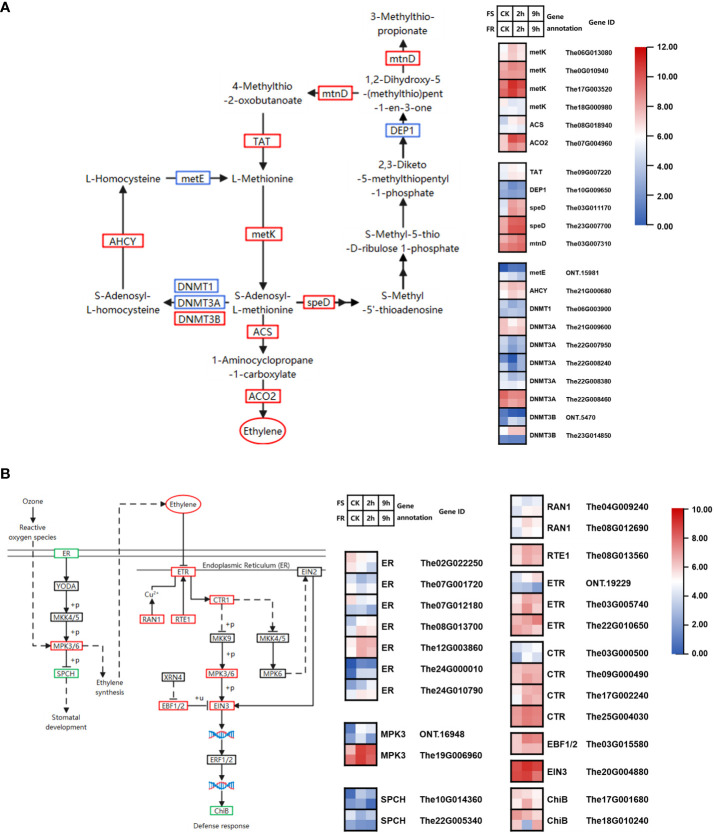
Cold-responsive DEGs regulate ethylene biosynthesis and signaling pathways. **(A)** Cold-responsive DEGs in ethylene biosynthesis. **(B)** Cold-responsive DEGs in the ethylene signaling pathway. Each colored cell in the heatmap represents log2 of the DEG CPM count values (mean of three biological replicates); red represents induced genes, and blue represents repressed genes. A schematic diagram of signaling pathway genes; red boxes represent all annotated genes that were upregulated, blue boxes represent all annotated genes that were downregulated, green boxes represent both up- and downregulated genes found in this location, and black boxes represent no DEGs found in this location.

We found 19 DEGs annotated to 10 KEGG catalogs in the methionine salvage pathway; six KEGG catalogues were upregulated, namely, *tyrosine aminotransferase (TAT), S-adenosylmethionine decarboxylase (speD), 1,2-dihydroxy-3-keto-5-methylthiopentene dioxygenase (mtnD), adenosylhomocysteinase (AHCY), cystathionine gamma-synthase (DNMT3B)*, and *S-adenosylmethionine synthetase (metK).* Among these, two *metK* genes *(The0G010940, The17G003520)* and two *speD* genes *(The03G011170, The23G007700)* were highly expressed and significantly induced, which may be key genes in promoting the methionine cycle (**Figure 6A**).

*1-Aminocyclopropane-1-carboxylate synthase (ACS)* and *aminocyclopropanecarboxylate oxidase (ACO2)* catalyze SAM to produce ethylene. *The08G018940* encoding *ACS* and *The07G004960* encoding *ACO2* were upregulated, especially the expression of *The07G004960* which was induced by 13.1-fold at 2 h and 8.2-fold at 9 h after cold treatment in FS and 3.9-fold at 2 h and 2.5-fold at 9 h after cold treatment in FR.

There were nine DEGs annotated to four KEGG catalogs in the ethylene synthesis pathway, including *ETR (ONT.19229, The03G005740 and The22G010650), CTR1 (The03G000500, The09G000490, The17G002240, and The25G004030), EIN3* (*The20G004880*), and *EBF (The03G015580)*. All DEGs were upregulated by cold stress. Among them, *EIN3* was highly expressed and significantly upregulated by cold stress (**Figure 6B**).

### 2.7 Validation of different expressed genes involved in cold response

**Figure 7:**
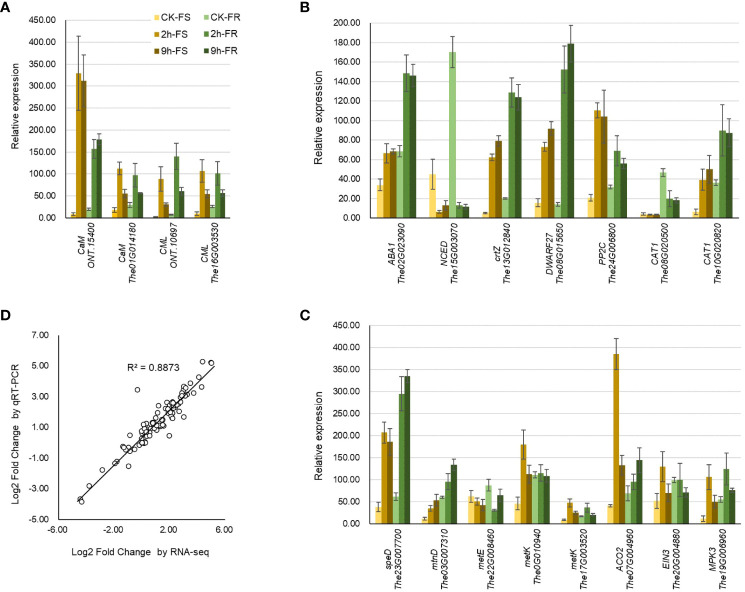
qRT-PCR analysis of different expressed genes involved in cold response. Relative expression of the key DEGs involved in **(A)** temperature sensing, biosynthesis, and signaling of **(B)** ABA and **(C)** ethylene. **(D)** Expression pattern validation by analyzing the linear dependence relation between the log2 fold change in key DEGs obtained from RNA-seq and qRT-PCR. The gene expression was determined by fold change relative to the reference gene *MDH*. All data were collected from three biological replicates and three technical replicates for each sample.

To verify the reliability and accuracy of RNA-seq data, quantitative real-time PCR (qRT-PCR) was used to detect the relative expression levels of 19 important and typical genes participating in temperature sensing, ABA, and ethylene biosynthesis and signaling pathways. Most of these genes were significantly and substantially induced by the 2-h and 9-h cold treatments (**Figures 7A–C**). The expression pattern detected with qRT-PCR was highly consistent with that detected using transcriptomics (R^2 =^ 0.8873) (**Figure 7D**); it revealed the reliability of these RNA-seq data.

### 2.8 Effect of ABA and ethylene on the *T. hemsleyanum* cold stress response

**Figure 8:**
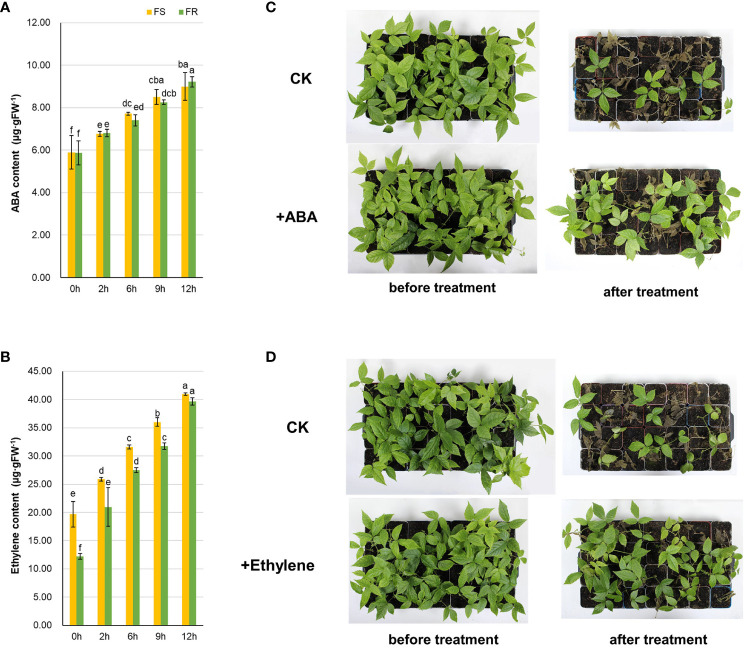
Effects of ABA and ethylene on cold tolerance in *T. hemsleyanum*. The variation in endogenous ABA **(A)** and ethylene **(B)** in FS and FR after chilling. Effect of exogenous ABA **(C)** and ethylene **(D)** on cold stress response after chilling. The content of plant hormones was detected after −2°C at different times, and data are the mean ± SD of three biological replicates. The letters indicate significant differences (multiple comparisons by Duncan, P < 0.05). The effect of exogenous phytohormones was tested in FS seedlings, and the phenotypes were recorded after −4°C treatment for 12 h.

Since we found that the expressions of key genes related to ABA and ethylene were induced in response to cold stress, the ABA and ethylene contents in *T. hemsleyanum* after simulated frost treatments were examined. Endogenous ABA and ethylene were both induced by chilling stress and increased during −2°C chilling (**Figures 8A, B**). There was no significant difference between ABA in FS and FR, whereas the ethylene level was higher in FS than in FR during a 12-h treatment. After 12 h of chilling, the ABA content increased by 52.6% in FS and 57.0% in FR whereas the ethylene increased more strongly, by 107.7% in FS and 225.0% in FR. The results also revealed the positive role of ABA and ethylene in response to chilling at the metabolic level.

In order to verify it further, exogenous phytohormones were sprayed on the FS seedlings, and −4°C chilling was applied. The chilling injury was obviously alleviated by ABA and ethylene, especially ethylene (**Figures 8C, D**). The survival rate was increased by 10.7% with ABA and 25.0% with ethylene.

## 3 Discussion

*Tetrastigma hemsleyanum* is an herb that is widely used in Chinese traditional and modern medicine. As a thermophilous plant, winter cold severely limits the artificial cultivation of this plant. Several studies have been carried out aimed at the cold response in *T. hemsleyanum*, but the mechanisms of cold sensitivity in this plant remain unclear. In this study, the transcriptomes in *T. hemsleyanum* seedlings of the cold-resistant variety FR and the cold-sensitive variety FS exposed to −2°C for 2 h and 9 h were compared and analyzed to decipher the *T. hemsleyanum* cold stress response.

A total of 76,750 transcripts and 28,805 genes were obtained, 4,121 of which were new genes; furthermore, the sequence was obtained using the latest ONT with exceptional read length, further enriching the existing *T. hemsleyanum* gene bank.

Calcium is a secondary messenger used in many plant signaling processes. Cell membrane solidification induced by cold can affect the structure and/or activity of membrane-localized proteins, thereby triggering Ca^2+^ influx, a crucial process for inducing temperature-responsive gene expression (Knight et al., 1996; Ding et al., 2020). Plants possessed several major systems to sense and conduct Ca^2+^ signaling, including CaM (calmodulin)/CMLs (CaM-like proteins), CCaMK (Ca^2+^- and Ca^2+^/CaM-dependent protein kinase), CDPKs (Ca^2+^-dependent protein kinases), and CBLs (calcineurin B-like proteins), CIPKs (CBL-interacting protein kinases) (Galon et al., 2010). In this research study, we found six genes encoding calmodulin proteins; four were induced by cold, and two were highly expressed, which may cause *OXI1* and three out of four *RbohD* genes to be upregulated in response to cold stress. In addition, we found 26 genes encoding CMLs, 25 induced by cold, and three were highly expressed. The results demonstrated that calcium signaling was regulated in response to the cold stress in *T. hemsleyanum*.

Except for the genes controlled by Ca^2+^, *RbohD* and *OXI1* are also regulated by *ROS* and *ROS*-generating stimuli independently, playing important roles in the plant immune response to pathogens. Specifically, *RbohD* regulates oxidative burst during perception of pathogen-associated molecular patterns (PAMPs) by pattern recognition receptors (PRRs), OXI1 kinase is required for activation of mitogen-activated protein kinases (MAPKs), and an essential part of the signal transduction pathway links oxidative burst signals to diverse downstream responses (Rentel et al., 2004; Kadota et al., 2015). In this research study, two out of three cold-induced *RbohD* genes were expressed more in FS, and *OXI1* was dramatically induced, and the expression in FS was 2.61-fold that in FR, which might cause more serious injury in FS after cold stress.

ABA is a vital phytohormone that regulates many essential physiological and biochemical processes, and it has a key role in stress resistance during plant growth and development (Huang et al., 2017). Stress rapidly triggers ABA production (Yamaguchi-Shinozaki and Shinozaki, 2006; Li et al., 2021), which causes ABA levels to increase and cold tolerance to be enhanced, such as in Arabidopsis and wheat (Choi et al., 2000; Hou et al., 2010). ABA is perceived by pyrabactin resistance (PYR)/PYR1-like (PYL)/regulatory components of ABA receptor (RCAR) receptors, which inactivate PP2C, resulting in activating the protein kinase SnRK2; SnRK2s activate many proteins via protein phosphorylation regulating stomatal closure (Lv et al., 2018). Group A PP2Cs interacted physically with SnRK2s in various combinations and efficiently inactivated ABA-activated SnRK2s via dephosphorylation of multiple Ser/Thr residues in the activation loop. This step was suppressed by the RCAR/PYR ABA receptors in response to ABA in *Arabidopsis* (Singh et al., 2015). The center stage in the ABA signaling pathway of PP2Cs was also verified in rice (Xue et al., 2008). In this research study, most DEGs in the ABA synthesis pathway were expressed at low levels or downregulated by cold, but genes encoding crtZ and ZEP were significantly induced by cold, which might be vital genes causing ABA to increase during short-term cold response in *T. hemsleyanum*. Some DEGs encoding PYL, PP2C, SnRK2, and ABF detected in this research study were also upregulated by cold, which may regulate numerous target genes in ABA-dependent pathways and ABA functional responses.

Ethylene is a crucial plant growth regulator that mediates cold stress responses in a species-dependent manner (Wang Y. C. et al., 2021). For example, ethylene positively affects the cold tolerance of tomato (*Lycopersicon esculentum*) (Ciardi et al., 1997). By contrast, ethylene levels are negatively correlated with the cold tolerance of Medicago truncatellid (Zhao et al., 2014). Low temperatures promote ethylene release in grapevines, and exogenous ACC (1-aminocyclopropane-1-carboxylat, the precursor of ethylene) increased the grapevine cold tolerance (Sun et al., 2016); ethylene positively regulated grapevine cold tolerance by modulating AP2/ERF and WRKY transcription factors, and the response of ABA and IAA during cold stress may be regulated by ETH signaling (Hou et al., 2023). *T. hemsleyanum* is also classified as a member of Vitisl. In this research study, DEGs encoding MPK3, which promoted ethylene synthesis, were upregulated, DEGs annotated to 12 KEGG catalogs in the ethylene synthesis pathway, and 10 KEGG catalogs were upregulated, including vital genes *ACS* and *ACO2*. DEGs annotated to four KEGG catalogs in the ethylene synthesis pathway were all upregulated by cold stress. It demonstrated that ethylene might be the major cold-responsive phytohormone within the 9 h stress treatment of *T. hemsleyanum.*

In addition, phytohormones can also regulate target genes through the MAPK signaling pathway. MAPK signaling is regulated by posttranslational modifications such as phosphorylation and ubiquitination, which might be the reason that we found no DEGs annotated to KEGG catalogs in some intermediate loci of transduction pathways, but DEGs encoding CAT were drastically changed. Posttranslational regulation might also happen in other nodes of phytohormone signaling pathways.

The 9-cis epoxycarotenoid dioxygenase (NCED) is considered to be a key rate-limiting enzyme in ABA biosynthesis (González-Silva et al., 2011); previous research has shown that cold stress caused significant upregulation of *NCED*s at 24 h in *T. hemsleyanum* (Peng et al., 2021). However, in this research study, two out of three *NCEDs* were suppressed by cold, and one out of three *NCEDs* were only significantly induced in FS. All *NCED* genes were expressed at low levels. It indicated that the 9-h cold treatment is not sufficient to activate some cold response methods; it is necessary to spend more time to observe long-term transcriptional regulations.

Many vital DEGs we found, such as *ONT.15400* (*CaM*), *The10G020820* (*CAT*), and *The19G006960* (*MPK3*), were expressed more in FR leaves at normal temperature, which might help form the cold tolerance. Some inherent traits that cannot be found in the transcriptome, such as the composition of the membrane, proline, and soluble sugar construction, might have reduced the cold injury before the cold response in the tissue of cold tolerance ecotypes, which may be the reason why more DEGs and more dramatic expression changes were found in the seedlings of sensitive ecotypes.

The change in expression level changed the endogenous ABA content, and exogenous ABA improved the cold tolerance of FS, consistent with previous research, which found endogenous ABA content at 0°C in other *T. hemsleyanum* ecotypes (Peng et al., 2021). Furthermore, we found a positive role of ethylene in response to cold stress, and the effect of ethylene might be stronger than ABA within 12 h.

## 4 Materials and methods

### 4.1 Plant materials and growth conditions

Two *Tetrastigma hemsleyanum* (Sanyeqing) ecotypes grown in the plant garden of Hangzhou Academy of Agricultural Sciences (Hangzhou, Zhejiang Province, China) were involved in this research study. FS has large leaves and stout semi-cylindrical stalks; FR has medium-sized leaves and medium-thickness, cylindrical stalks.

Stem segments with one node and one bud were clipped and planted in peat. After 2 months of growth, cutting seedlings that had built root systems and sprouted five to six nodes were selected.

### 4.2 RNA isolation and transcriptome sequencing

#### 4.2.1 Library preparation

Selected seedlings were exposed to a simulated frost treatment (−2°C) for 0 h, 2 h, and 9 h. Three samples were performed for each cultivar, and each sample contained 12 plants. Leaves at the third and fourth nodes from the tip were collected and stored at −80°C for further analysis.

RNA samples were prepared using an RNA simple Total RNA Kit (DP411, TIANGEN), RNA integrality was tested by agarose gel (LabChip GX, Agient2100), and the RNA concentration was detected using a NanoDrop 2000 (Thermo Fisher Scientific).

Library preparation was performed according to the standard protocol provided by ONT. A mass of 1 μg total RNA was prepared for cDNA libraries using the cDNA-PCR Sequencing Kit (SQK-LSK110+EXP-PCB096) protocol provided by ONT. The template-switching activity of reverse transcriptases enriches full-length cDNAs and adds defined PCR adapters directly to both ends of the first-strand cDNA, followed by cDNA PCR for 14 cycles with LongAmp Tag (NEB). The PCR products were then subjected to ONT adaptor ligation using T4 DNA ligase (NEB). Agencourt XP beads were used for DNA purification according to the ONT protocol. The final cDNA libraries were added to the PromethION Flow Cell (R9 Version, FLO-PRO002, Nanopore) and run on the PromethION platform at Biomarker Technology Company (Beijing, China).

#### 4.2.2 Data optimization

Raw reads were first filtered with a minimum average read quality score = 6 and a minimum read length = 350 bp. Ribosomal RNA was discarded after mapping to the rRNA database. Next, full-length, non-chimeric (FLNC) transcripts were determined by searching for primers at both ends of the reads. Clusters of FLNC transcripts were obtained after mapping to the reference genome with mimimap2 (2.16), and consensus isoforms were obtained after polishing within each cluster by pinfish (v0.1.0).

#### 4.2.3 Quantification of gene/transcript expression levels and differential expression analysis

Consensus sequences were mapped to the reference genome using minimap2 (2.16). Mapped reads were further collapsed by the cDNA_Cupcake (5.8) package with min-coverage = 85% and min-identity = 90%. A 5′ difference was not considered when collapsing redundant transcripts. The coding sequence (CDS) was predicted using TransDecoder (v3.0.0).

Quantification of gene/transcript expression levels and differential expression analysis

Full-length reads were mapped to the reference transcriptome sequence. Reads with a match quality above 5 were used to quantify further. Expression levels were estimated by reads per gene/transcript per 10,000 mapped reads. For samples with biological replicates, differential expression analysis of two conditions/groups was performed using the DESeq2 R package (1.6.3) (Love et al., 2014). DESeq2 provides statistical routines for determining differential expression in digital gene expression data using a model based on the negative binomial distribution. The resulting P values were adjusted using Benjamini and Hochberg’s approach for controlling the FDR. Genes with an FDR <0.05 and fold change ≥2 found by DESeq2 were assigned as differentially expressed. Heatmaps were generated by TBtools (https://github.com/CJ-Chen/TBtools).

#### 4.2.4 Gene functional annotation and enrichment analysis

Gene Ontology (GO) enrichment analysis was implemented using the GOseq R (2.18.0) packages based on Wallenius non-central hyper-geometric distribution (GO database released on 2020-06-01) (Young et al., 2010). KOBAS (Mckenna et al., 2010) software was used to test the statistical enrichment of DEGs in KEGG pathways (KEGG version is 20191220). The obtained novel transcript sequences were also aligned to the NR (202009) (Deng et al., 2006), Swissprot (202005) (Apweiler et al., 2004), COG (COG2014) (Tatusov et al., 2000), KOG (KOG2003) (Koonin et al., 2004), and Pfam (Pfam33.1) (Kanehisa et al., 2004) databases. GSEA was performed using online tools in BMKCloud (www.biocloud.net).

#### 4.2.5 WGCNA analysis

WGCNA with all 18 libraries acquired from ONT RNA-seq was performed using the WGCNA R package in BMKCloud (www.biocloud.net) (Langfelder and Horvath, 2008). Co-expression network modules were identified based on counts per million mapped reads (CPM) ≥10, variation of CPM ≥0.5; the minimum module size was 10, the minimum height for merging modules was 0.3, and the soft threshold power was picked by the program automatically. The central hub genes were defined as those with module eigengene-based connectivity (kME) >0.7 within an assigned module, and modules were correlated with ecotypes and treatments to identify co-expression.

### 4.3 Validation of gene expressions involved in cold response

Plant tissue was the same batch as that used for transcriptome sequencing. RNA samples were prepared using the RNeasy Plant Mini Kit (Qiagen), and DNA was eliminated using an RNase-Free DNase Set (Qiagen). cDNAs were synthesized using SuperScript™ III First-Strand Synthesis SuperMix for qRT-PCR (Invitrogen). The samples were amplified in Power SYBR^®^ Green PCR Master Mix (Applied Biosystems) and detected using a CFX384 Real-Time PCR System (Bio-Rad). Three samples were performed for each cultivar, and three replicates were performed for each sample. The *MDH (malic dehydrogenase)* gene was selected as an internal reference gene. The information on DEGs whose expression have been detected is offered in **Supplementary Table 2**. The primers were designed using Beacon Designer 7.8 and Primer Premier 6.0 (**Supplementary Table 3**).

### 4.4 Phytohormone measurement and exogenous phytohormone treatment

Plant samples used for phytohormone measurement were prepared as described in 2.1. A mass of 0.1g tissue from each biological replicate was crushed in 1 mL of PBS solution (0.01 mol·L^−1^, pH = 7.2–7.4). The measurement was performed according to the standard protocol of ELISA kits provided by mlbio Biotechnology (https://www.mlbio.cn/).

Plants used for evaluation of the effect of exogenous phytohormones were cultivated as described in 4.1. A total of 28 seedlings were included for each treatment; 100 mL of 500 μmol/L ABA or 60 mg/L ethylene was sprayed on seedlings and cultivated for 3 days and then sprayed with exogenous phytohormones and cultivated for 3 days again. Treated seedlings were exposed to a −4°C simulated frost treatment for 12 h.

## Data availability statement

The original data of ONT transcriptome presented in the study are deposited in the database Genome Sequence Archive (GSA) of CNCB-NGDC, accession number SRR14575715-SRR14575720 under bioproject accession PRJCA016284. Other data presented in this study can be found in **Supplementary Material**.

## Author contributions

LQ: Conceptualization, Funding acquisition, Methodology, Project administration, Resources, Supervision, Writing – original draft, Writing – review & editing. SY: Data curation, Investigation, Visualization, Writing – original draft, Writing – review & editing. NL: Investigation, Writing – review & editing. EY: Writing – review & editing. JY: Funding acquisition, Project administration, Resources, Writing – review & editing.
