# Supplementary material for: Full-length transcriptome reveals the pivotal role of ABA and ethylene in the cold stress response of Tetrastigma hemsleyanum
Source: Front Plant Sci. 2024 Jan 31;15:1285879. doi: 10.3389/fpls.2024.1285879 (PMC10864657; doi:10.3389/fpls.2024.1285879)
Supplement: Supplementary file 1 [file DataSheet_1.docx]

| A  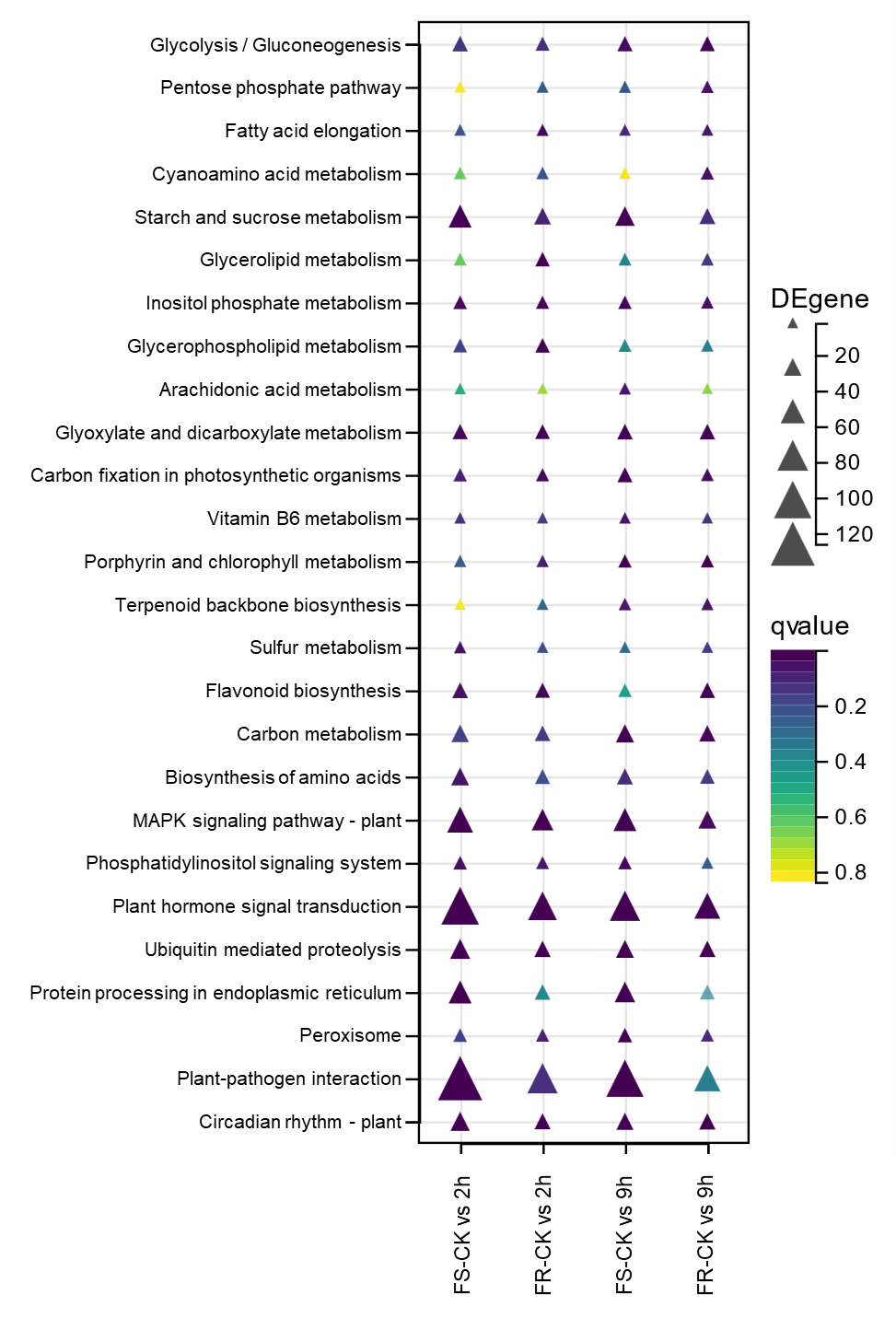 | B  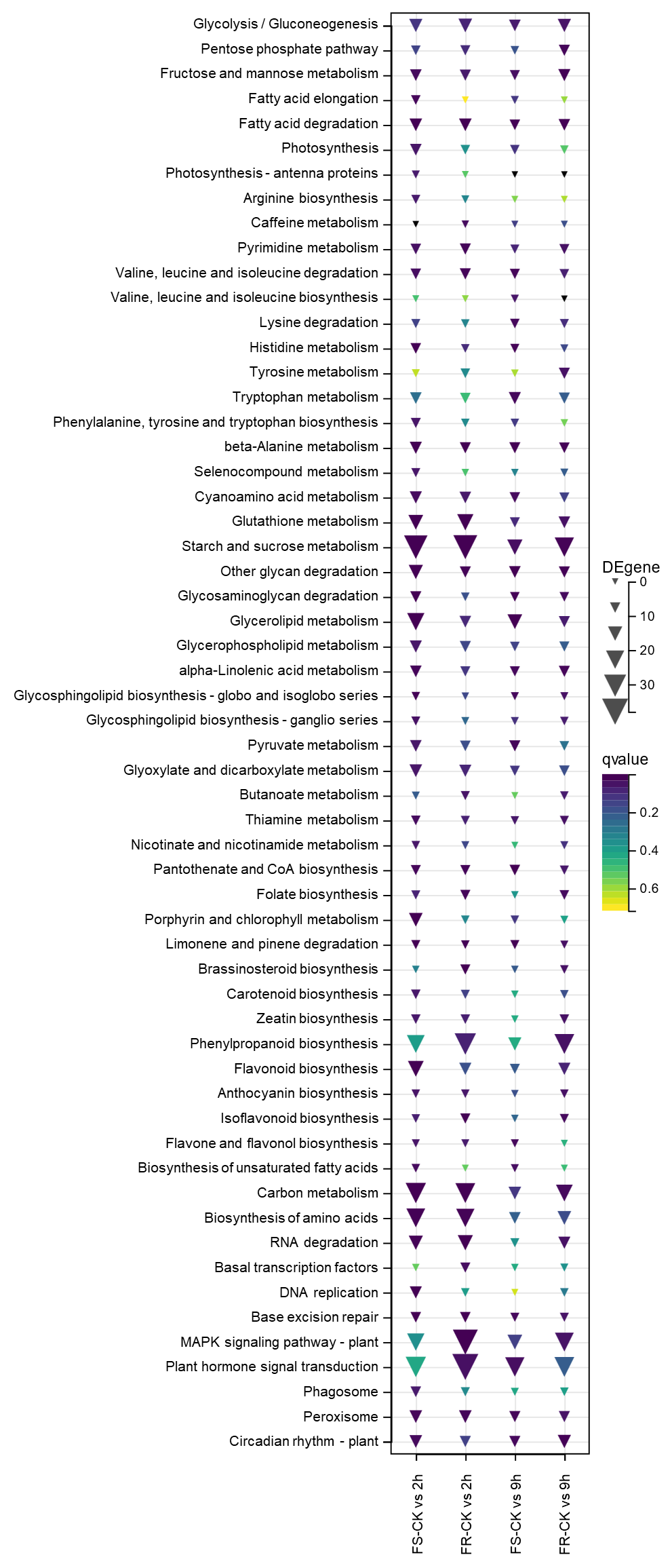 |
| --- | --- |

**Supplementary Figure 1.** KEGG pathway enrichment analysis based on DEGs expressed (A) induced and (B) suppressed by cold compare with controls..
